# Effects of Introduced and Indigenous Viruses on Native Plants: Exploring Their Disease Causing Potential at the Agro-Ecological Interface

**DOI:** 10.1371/journal.pone.0091224

**Published:** 2014-03-12

**Authors:** Stuart J. Vincent, Brenda A. Coutts, Roger A. C. Jones

**Affiliations:** 1 Department of Agriculture and Food, South Perth, Western Australia, Australia; 2 State Agricultural Biotechnology Centre, School of Biological Sciences and Biotechnology, Murdoch University, Murdoch, Western Australia, Australia; 3 School of Plant Biology, Faculty of Science, The University of Western Australia, Crawley, Western Australia, Australia; Washington State University, United States of America

## Abstract

The ever increasing movement of viruses around the world poses a major threat to plants growing in cultivated and natural ecosystems. Both generalist and specialist viruses move via trade in plants and plant products. Their potential to damage cultivated plants is well understood, but little attention has been given to the threat such viruses pose to plant biodiversity. To address this, we studied their impact, and that of indigenous viruses, on native plants from a global biodiversity hot spot in an isolated region where agriculture is very recent (<185 years), making it possible to distinguish between introduced and indigenous viruses readily. To establish their potential to cause severe or mild systemic symptoms in different native plant species, we used introduced generalist and specialist viruses, and indigenous viruses, to inoculate plants of 15 native species belonging to eight families. We also measured resulting losses in biomass and reproductive ability for some host–virus combinations. In addition, we sampled native plants growing over a wide area to increase knowledge of natural infection with introduced viruses. The results suggest that generalist introduced viruses and indigenous viruses from other hosts pose a greater potential threat than introduced specialist viruses to populations of native plants encountered for the first time. Some introduced generalist viruses infected plants in more families than others and so pose a greater potential threat to biodiversity. The indigenous viruses tested were often surprisingly virulent when they infected native plant species they were not adapted to. These results are relevant to managing virus disease in new encounter scenarios at the agro-ecological interface between managed and natural vegetation, and within other disturbed natural vegetation situations. They are also relevant for establishing conservation policies for endangered plant species and avoiding spread of damaging viruses to undisturbed natural vegetation beyond the agro-ecological interface.

## Introduction

Long before plants were first domesticated at the dawn of agriculture, viruses were evolving with native plants growing within undisturbed plant communities in different regions of the world. This evolutionary process moulded both viruses and native plants [1], [2], [3]. Within the undisturbed native plant communities of today, virus infections derived from this process are often considered benign, causing little in the way of damage or symptoms due to a combination of natural control measures that operate to limit epidemics and the consequences of evolution with their hosts over millennia [2]–[13]. However, this is not always so as virus infections sometimes cause obvious damage to native plants growing within undisturbed natural vegetation [2], [5], [14], [15]. Also, the abundance of virus resistance genes in wild ancestors of modern crop plants provides evidence of past battles between them and viruses that evolved with them [2]. Moreover, in mixed native plant species populations, viruses can impact on community structure and dynamics by decreasing the competitive and reproductive abilities of infected plants [2], [16]–[19]. Even mild virus symptoms in one species in a mixed plant species population containing host and non-host species can alter its competitive ability sufficiently to alter plant species composition [2], [20]–[24]. Also, mild infection in one host species in a mixed plant species population can provide a virus reservoir for spread to more sensitive host species, and, once infected, these decline due to lack of fitness and competitive ability [25], [26].

Long before the dawn of agriculture, generalist viruses are considered to have evolved in species-rich native plant communities where they infected a wide range of hosts belonging to different plant families. In contrast, specialist viruses are considered to have evolved first in native plant communities with few species and relied on just a few natural hosts. Both generalist and specialist plant viruses still occur in undisturbed natural vegetation. They also occur in cultivated plants in which they often cause virus epidemics [2], [3], [6]–[9]. A decision over whether a virus is a generalist or specialist is based on its known natural host range and not on the extent of its host range when plants are inoculated under glasshouse conditions [3], [6], [7], [27]. The process of wild plant domestication can favour selection of specialist host-adapted strains within generalist viruses and new specialist viruses [28]–[30]. Genomic divergence is roughly proportional to the evolutionary distance from a common ancestor [31], and a high degree of sequence diversity over a small geographic range is typical of viruses that evolved with native plants locally over a long period of time [15], [32]. Such viruses are referred to as indigenous to distinguish them from others that arrived from elsewhere recently and therefore show only low local sequence diversity, for which the term introduced is used [3], [15], [33].

New encounter scenarios involving viruses and plant species are becoming increasingly common because of rapidly increasing human activity, such as agricultural practices involving extensification, intensification and diversification, necessitated by the need to maintain food security and feed the burgeoning human population [3], [7], [34]–[37]. In the future, their frequency is expected to increase even faster because of the major alterations in crop distributions anticipated from global warming associated with climate change [3], [38]. When new encounters between introduced viruses and native plants occur, such viruses can invade native plant species they have not met previously. Also, the natural control measures which help to diminish virus spread in undisturbed native plant communities, such as isolation, admixture with non-hosts, host resistance/tolerance and presence of predators and parasites of their vectors, are disrupted when such communities are disturbed, as occurs at the agro-ecological interface between cultivated and natural vegetation and in otherwise disturbed natural vegetation [3], [6], [7], [9]. Because the simplicity of plant virus genomes allows generalist viruses to adapt quickly to new hosts, an increased frequency of virus invasion of native plants at this interface and their subsequent spread beyond it to undisturbed native plant communities is cause for concern for survival of endangered native plant species [3], [38].

The Southwest Australian Floristic region (SWAFR) is a species rich global bio-diversity hot spot [39], [40] with *c*.8,000 native plant species, 49% of which are endemic and *c.*2,500 are endangered by human encroachment and climate change. It occupies 302,672 m^2^ and is isolated from the rest of Australia by deserts. It has a harsh Mediterranean-type climate and some of the world's oldest, weathered, infertile landscapes [41]. It is unique geographically because of its isolation, geological stability and absence of human disturbance until recently. No plants were cultivated in the SWAFR before European colonisation in 1829 so its interfaces between recent managed and ancient natural vegetation are ideal for distinguishing introduced from indigenous viruses. In parts of the world where plants have been cultivated for much longer, making this distinction becomes more difficult [15], [36]. The SWAFR's vegetation interfaces are also well suited for studying the likely impact of newly introduced viruses on species dynamics in communities of native plants.

In the SWAFR, virus-like foliage symptoms have been observed for many years occurring commonly in native plants growing at the managed and ancient natural vegetation interface. For example, a mosaic disease of the perennial native legume *Kennedia prostrata* was described in 1956 [42]. When samples from native plants with leaf symptoms were tested, McKirdy et al. [43] identified the introduced generalist virus, *Bean yellow mosaic virus* (BYMV), infecting *K. prostrata* plants with mosaic and stunting symptoms, and the perennial native legume species, *K. coccinea, Hovea elliptica* and *H. pungens*. They also recorded obvious foliar virus symptoms (yellow mottles) without identifying potentially causal viruses in several other native legumes, including *Bossiaea eriocarpa*, *Erythrina indica*, *Hardenbergia comptoniana* and *Hovea chorizemifolia*. In other studies, the introduced specialist viruses *Barley yellow dwarf virus* (BYDV) and/or *Cereal yellow dwarf virus* (CYDV) were identified infecting four native grass species: both viruses in *Chloris truncata* and *Pennisetum alopecuroides*, and CYDV alone in *Microlaena stipoides* and *Themeda australis* [44]–[46]. The recently introduced specialist virus *Wheat streak mosaic virus* (WSMV) was found symptomlessly infecting the native grass species *Tragus australianus* [47]. The introduced generalist virus *Tomato spotted wilt virus* (TSWV) was detected symptomlessly infecting a native species in the Dasypogon family, *Calectasia cyanea* [48], and the introduced generalist viruses BYMV, *Turnip yellows virus* (TuYV) and *Ornithogalum mosaic virus* (OrMV) were found infecting native orchid plants (*Diurus* spp.) sometimes showing foliar virus symptoms [49]. However, despite such findings of introduced viruses infecting native plants, the potentially damaging impact that recently introduced plant viruses may have on native plant species biodiversity in the SWAFR and elsewhere continues to be largely ignored. This is mainly due to attribution of their foliar symptoms to other causes, such as nutritional deficiencies.

Previous studies of virus distribution and occurrence in introduced cultivated plants in the SWAFR revealed many virus infection reservoirs from which introduced generalist viruses could invade native plants including (viruses commonly present in square brackets): annual clover and medic pastures [*Alfalfa mosaic virus* (AMV), BYMV]; perennial alfalfa and white clover pastures [AMV, TuYV]; grain legume crops [AMV, BYMV, *Cucumber mosaic virus* (CMV), TuYV]; infected oilseed crops [*Turnip mosaic virus* (TuMV), TuYV]; and vegetables such as tomato and pepper [CMV, TSWV, TuYV] [21]–[23], [43], [50]–[60]. Infection reservoirs from which introduced specialist viruses can spread from introduced cultivated plants to invade native plants growing at the interface include: perennial ryegrass in pastures [BYDV, *Ryegrass mosaic virus* (RyMV)], cereals [BYDV, CYDV, WSMV], field pea [*Pea seed-borne mosaic virus* (PSbMV] and potato [*Potato virus X* (PVX), *Potato virus S* (PVS)] [21], [44], [48], [52], [61], [62]. Moreover, introduced viruses that occur naturally in introduced weeds in the SWAFR include six generalists [AMV, BYMV, CMV, TSWV, TuMV, TuYV] and three specialists [BYDV, CYDV, WSMV] [22]–[24], [43], [45]–[48], [53], [60], [61], [63].

Examples of viruses that are indigenous to Australia [64], [65] and occur in the state of Western Australia, where the SWAFR is located, include the potyviruses, Clitoria chlorosis virus (ClCV), *Hardenbergia mosaic virus* (HarMV), *Passiflora virus Y* (PaVY) and *Passionfruit woodiness virus sensu stricto* (PWV) [15], [33]. Of these, HarMV is indigenous to, and PWV occurs within, the SWAFR [15] while PWV, ClCV and PaVY are found further north in the state [33]. Further information on their natural host ranges is required before any of them can be assigned to specialist or generalist categories. Several other viruses recently found infecting native plants using deep sequencing methodologies may be indigenous to the SWAFR [49], [66], [67].

Our aim was to study the effects of introduced and indigenous viruses on native plants by exploring their disease causing potential at the agro-ecological interface. With the exception of the indigenous viruses ClCV and PaVY found elsewhere in the State and *Barley stripe mosaic virus* (BSMV) found locally in barley germplasm [68], the spectrum of viruses tested was based entirely on ones known to occur in the SWAFR. Our approach was to compare the impacts of six introduced generalist [AMV, BYMV, CMV, TSWV, TuMV, TuYV], seven introduced specialist [BSMV, BYDV, RyMV PSbMV, PVX, PVS, WSMV] and four Australian indigenous viruses [ClCV, HarMV, PaVY, PWV] on the appearance, growth and viability of a selection of native plant species from the SWAFR belonging to eight families, and provide additional information on the occurrence of introduced viruses in native plants at the agro-ecological interface in the region. The hypotheses tested were that (i) introduced generalist and indigenous viruses are likely to cause severe symptoms and growth reductions when they invade native species they have not encountered before, (ii) specialist introduced viruses are less likely to damage native plants severely, and (iii) infections with introduced generalist viruses are becoming increasingly widespread in natural vegetation at the agro-ecological interface in the SWAFR. The results obtained provided evidence supporting all three of these hypotheses.

## Materials and Methods

### Ethics statement

Native plant leaf samples were collected under Western Australian Department of Parks and Wildlife flora licences or with permission from Kings Park Botanic Gardens. None of the species sampled in the field or the 15 native plant species grown for virus inoculations were endangered. The only endangered species sampled was a plant of the native orchid *Thelymitra* sp. which was being propagated in a secure glasshouse at Kings Park Botanic Gardens for return to the wild (http://florabase.dpaw.wa.gov.au). None of the field sites sampled was privately owned except for the site at Wellard which belongs to the paper's second author (BAC).

### Glasshouse grown plants, inoculations, virus isolates and antibodies

All virus culture hosts and the native plants used in the experiments were grown in insect-proof, air-conditioned glasshouses maintained at 18–22°C. Seed of culture hosts was sown in a steam-sterilized potting mix containing soil, sand and peat (1∶1∶1). Seed of native plants used in the experiments was obtained from commercial suppliers or Department of Agriculture and Food Western Australia (DAFWA) Floriculture projects. Seeds of four native legume species were soaked in boiling water for 24 hours to break dormancy. These soaked legume seeds and the seeds of six other native plant species belonging to other families were sown in native plant potting mix (Flori mix No. 2, SSM0015, RichGrow) mixed with granulated polystyrene (2∶1) in covered trays. The trays were placed onto heated sand beds in a misting cabinet until germination when the seedlings were transplanted into pots containing more native plant potting mix. Healthy plants of two other native species (*Anigozanthos manglesii* and *A. flavidus*) were subdivided into smaller plants which were planted in native plant potting mix in pots and kept in a misting cabinet until they had rooted. Three other native species (*K. coccinea*, *H. comptoniana* and *Hibbertia cuneformis*) were propagated from healthy shoot cuttings by dipping these in propagation gel (3 g/L indole 3-Butyric acid) and planting in punnets in a mix of peat, granulated polystyrene and pearlite (1∶1∶1). The cuttings were kept in a misting cabinet until they rooted before being transplanted to pots.

Tables 1 and 2 list the 13 introduced and four indigenous viruses used, their acronyms, classifications, modes of transmission, host specificities, codes used to distinguish isolates, original isolate references, culture hosts used for each virus isolate, virus antibodies used in enzyme-linked immunosorbent assay (ELISA) and names of antibody suppliers. Infected culture hosts acted as sources for five introduced generalist, seven introduced specialist and two indigenous viruses. One introduced generalist virus (AMV) was sourced from infected seedlings growing from an infected seed stock of its culture host. The sources of two indigenous viruses (HarMV, PWV) were virus-infected perennial hosts growing outside at the DAFWA South Perth site. Plants of virus culture hosts used in the glasshouse and of the 15 native plant species used in the experiments were inoculated with sap or aphids, and one species was also inoculated by grafting. Sap inoculations were done by grinding infected leaves in 0.05 M phosphate buffer, pH 7.2, with 0.01 M sodium sulphite, and the sap extract was then mixed with celite before being rubbed onto leaves. For aphid inoculations, *Myzus persicae* (green peach aphid) or *Rhopalosiphum padi* (oat aphid) were placed on virus-infected leaves for 2 days, transferred to healthy plants (10 aphids/plant) for 1–2 days and then killed with aphicide. For graft inoculations, shoots cut from virus-infected plants were top-grafted onto healthy plants.

**Table 1:** Introduced and indigenous viruses used: classifications, modes of transmission, host specificities and isolates.

Virus (acronym)	Genus	Family	Type of vector transmission	Host specificity	Isolate(s) used	Isolate reference(s)
**Introduced virus**						
*Alfalfa mosaic virus* (AMV)	*Alfamovirus*	*Bromoviridae*	Aphid (NP)^A^	Generalist	EW	Jones and Pathipanawat [55]
*Barley stripe mosaic virus* (BSMV)	*Hordeivirus*	*Virgaviridae*	Contact	Specialist	ES1	This study
*Barley yellow dwarf virus* (BYDV)	*Luteovirus*	*Luteoviridae*	Aphid (P)	Specialist	PAV-Manj1 (PAV-M1, 2001)	This study (M1, McKirdy and Jones [45])
*Bean yellow mosaic virus* (BYMV)	*Potyvirus*	*Potyvirideae*	Aphid (NP)	Generalist	MI, FB1	MI, Jones [88]; FB1,Cheng and Jones [89])
*Cucumber mosaic virus* (CMV)	*Cucumovirus*	*Cucumoviridae*	Aphid (NP)	Generalist	LW, SN	Jones [53]
*Pea seed-borne mosaic virus* (PSbMV)	*Potyvirus*	*Potyvirideae*	Aphid (NP)	Specialist	W1	Latham and Jones [52]
*Potato virus S* (PVS)	*Caralvirus*	*Betaflexiviridae*	Contact, Aphid (NP)	Specialist	SK	Wilson and Jones [90]
*Potato virus X* (PVX)	*Potexvirus*	*Alphaflexiviridae*	Contact	Specialist	XK	Wilson and Jones [91]
*Ryegrass mosaic virus* (RyMV)	*Rymovirus*	*Potyviriadae*	Eriophyid mite	Specialist	AV1	Coutts and Jones [21]
*Tomato spotted wilt virus* (TSWV)	*Tospovirus*	*Bunyaviridae*	Thrips	Generalist	Crb1, LT (CaWA1, 2001)	This study (CaWA1, Latham and Jones [48]
*Turnip yellows virus* (TuYV)	*Polerovirus*	*Luteoviridae*	Aphid (P)	Generalist	WA1	Coutts and Jones [59]
*Turnip mosaic virus* (TuMV)	*Potyvirus*	*Potyviridae*	Aphid (NP)	Generalist	WA-Ap1	Coutts and Jones [59]
*Wheat streak mosaic virus* (WSMV)	*Tritimovirus*	*Potyviridae*	Eriophyid mite	Specialist	Mer1, Gin	Mer1 (Coutts et al. [61]); Gin (Dwyer et al. [92];
**Indigenous virus**						
Clitoria chlorosis virus (ClCV)	*Potyvirus*	*Potyviridae*	Aphid (NP)	Not determined	13B	Coutts et al. [33]
*Hardenbergia mosaic virus* (HarMV)	*Potyvirus*	*Potyviridae*	Aphid (NP)	Not determined	Cgt, SP1	Webster et al. [15]
*Passiflora virus Y* (PaVY)	*Potyvirus*	*Potyviridae*	Aphid (NP)	Not determined	CarP1, KnxP1, KnxP5	Coutts et al. [33]
*Passionfruit woodiness virus* (PWV)	*Potyvirus*	*Potyviridae*	Aphid (NP)	Not determined	SP1	Coutts et al. [33]

A P =  Persistently transmitted; NP  =  Non Persistently transmitted

**Table 2:** Introduced and indigenous viruses used: culture hosts for each isolate and antibodies for detection.

Virus	Isolate	Culture host	Antiserum used	Antiserum source
**Introduced**				
*Alfalfa mosaic virus* (AMV)	EW	*Medicago polymorpha* cv. Serena (burr medic)^A^	AMV	DSMZ
*Barley stripe mosaic virus* (BSMV)	ES1	*Hordeum vulgare* cv. Stirling (barley)	BSMV	Loewe
*Barley yellow dwarf virus* (BYDV)	PAV-Manj1 (PAV-M1, 2001)	*Hordeum vulgare* cv. Stirling (barley), or *Triticum aestivum* cvs Calingiri or Spear (wheat)	BYDV-PAV	Loewe
*Bean yellow mosaic virus* (BYMV)	FB1	*Vicia faba* cv. Fiord (faba bean)	BYMV	DSMZ
*Bean yellow mosaic virus* (BYMV)	MI	*Trifolium subterraneum* cv. Woogenellup (subterranean clover)	BYMV	DSMZ
*Cucumber mosaic virus* (CMV)	LW	*Lupinus angustifolius* cv Wandoo (narrow-leafed lupin)	CMV	Loewe
*Cucumber mosaic virus* (CMV)	SN	*Trifolium subterraneum* cv. Woogenellup (subterranean clover)	CMV	Loewe
*Pea seed-borne mosaic virus* (PSbMV)	W1	*Vicia faba* cv. Fiord (faba bean)	PSbMV	DSMZ
*Potato virus S* (PVS)	SK	*Solanum tuberosum* cv. Ruby Lou (potato)	PVS	Loewe
*Potato virus X* (PVX)	SX	*Solanum tuberosum* cv. Eben (potato)	PVX	Loewe
*Ryegrass mosaic virus* (RyMV)	AV1	*Lolium perrene* cv. Unknown (perennial ryegrass)	Generic potyvirus,	Agdia
*Tomato spotted wilt virus* (TSWV)	Crb1, LT (CaWA1, 2001)	*Lycopersicon esculentum* cv. Grosse Lisse (tomato)	TSWV, Generic tospovirus	Loewe
*Turnip yellows virus* (TuYV)	WA1	*Brassica napus* cv. Pinnacle (canola)	BWYV^B^	Loewe
*Turnip mosaic virus* (TuMV)	WA-Ap1	*Brassica juncea* cv. Tendergreen (Indian mustard)	TuMV	DSMZ
*Wheat streak mosaic virus* (WSMV)	Gin, Mer1	*Triticum aestivum* cv. Calingiri (wheat)	WSMV	Loewe
**Indigenous**				
Clitoria chlorosis virus (ClCV)	13B	*Nicotiana benthamiana*	BCMV^B^	Loewe
*Hardenbergia mosaic virus* (HarMV)	Cgt	*Hardenbergia violacea* (native lilac)^A^	Generic potyvirus	Agdia
*Hardenbergia mosaic virus* (HarMV)	SP1	*Hardenbergia comptoniana* (native wisteria)^A^	Generic potyvirus	Agdia
*Passiflora virus Y* (PaVY)	CarP1	*Nicotiana benthamiana*	Generic potyvirus	Agdia
*Passiflora virus Y* (PaVY)	KnxP1, KnxP5	*Macroptilium atropurpureum* (siratro)	Generic potyvirus	Agdia
*Passionfruit woodiness virus* (PWV)	SP1	*Passiflora coerulea* (blue passion flower)^A^	Generic potyvirus	Agdia

Virus specific polyclonal antisera always used in ELISA tests on samples, except for generic potyvirus monoclonal antibodies from Loewe (inoculations to native species, and 2001+2009 field samples), tospovirus serogroups I, II and III from Sanofi (2001+2009 field samples), and luteovirus (1991 field samples) from DSMZ. In addition, polyclonal antiserum to *Impatiens necrotic spot virus* (INSV) from DSMZ was used once in 2009.

A These virus source plants were growing outside at DAFWA, South Perth either in hedges (PWV and HarMV-SP1) or as a potted plant (HarMV-Cgt), or in the glasshouse from infected seedlings grown from an infected seed stock (AMV-EW).

B These polyclonal antibodies had broad specificity, also detecting related viruses, BCMV  =  *Bean common mosaic virus*, BWYV * =  Beet western yellows virus*.

### Enzyme-linked immunosorbent assay

Leaf or flower samples were extracted (1 g 20ml^−1^) in phosphate buffered saline (10 mM potassium phosphate, 150 mM sodium chloride), pH 7.4, containing 5 ml litre^−1^ of Tween 20 and 20 g litre^−1^ of polyvinyl pyrrolidone using a leaf press (Pollahne, Germany). The extracts were collected in labelled, plastic sample tubes and tested by double antibody sandwich ELISA using paired wells in immunoplates as described by Clark and Adams [69]. To detect potyvirus infection, leaf or flower samples were extracted (1 g/20 ml) in carbonate buffer and tested using the antigen-coated indirect ELISA protocol described by Torrance and Pead [70]. For both types of ELISA, each sample was tested in duplicate wells in microtitre plates and appropriate infected and healthy leaf samples were included in paired wells as controls. BYMV-, TuYV- or TSWV-infected sap was used as the positive control for the potyvirus, luteovirus or tospovirus monoclonal antibodies, respectively.

### Experiments

Eleven native plant species were inoculated with 11–12 viruses and four with 3–7 viruses (Tables 1 and 2). The same 10 viruses were inoculated to each of the 11 species: five introduced generalists (AMV, BYMV, CMV, TSWV, TuMV), two introduced specialists (PSbMV and PVX) and three indigenous viruses (ClCV, HarMV, PWV). The generalist virus TuYV and introduced virus PaVY were inoculated to nine of these species each. Specialist virus PVS was inoculated to one of them, and the specialists BSMV, BYDV, RyMV and WSMV were inoculated to a native grass species which was not one of the 11 species. In the inoculations, each virus isolate used was inoculated to at least five plants of each species and also to a plant of a standard indicator host for the virus, and another five plants were mock-inoculated with healthy leaf sap. Any symptoms that developed in the inoculated plants were recorded over a 6 week period. The indicator host was included as a positive control to confirm that the inoculum used was sufficiently infective to cause infection. The mock-inoculated plants served as a healthy control for comparison with plants that developed virus symptoms. Samples from inoculated and tip leaves of each sap inoculated plant were tested for infection with the virus inoculated to them by ELISA. Samples from inoculated or tip leaves were grouped separately initially for each virus-plant species combination, but if virus infection was detected each sample was tested individually. Where plants were inoculated by aphids or grafting, only samples from tip leaves were tested.

### Effects on plant growth and biomass

Plants kept from some of the inoculations described in the previous section were re-potted in large pots in native plant soil mix for use in small-scale experiments. For each of nine virus-host combination, the pots were then arranged on the glasshouse bench to provide alternating pairs of healthy and virus-infected plants. The plants were staked and fertilised as required. Apart from *Solanum symonii* (Solanaceae), all the native plant species used always remained vegetative for a long period under glasshouse conditions instead of entering a reproductive phase. When they were mature, or still had not produced any fruits or pods up to 8 months after inoculation, the foliage from each plant was cut off at ground level, bagged separately, dried and then weighed. The individual plant foliage dry weight data from the pairs of plants were then subjected to a t-test.

In two experiments with *S. symonii*, the pots were arranged on the glasshouse bench in randomised block designs. There were four treatments with four replicates each in the first experiment and three treatments with five replicates each in the second. In the first experiment, the treatments were mock-inoculated control plants and plants inoculated with TSWV-Crb1, CMV-SN or both AMV and CMV-SN. In the second experiment, they were mock-inoculated control plants and plants inoculated with TSWV-Crb1 or AMV. The foliage from each plant was cut off at ground level, bagged separately, dried and weighed as described above. Also, in the first experiment the fruits were collected from each plant, bagged separately from the foliage, dried and weighed. Then, the dried fruits from each plant were counted and a mean individual fruit weight per plant calculated by dividing total fruit weight by numbers of fruits. The foliage and fruit dry weight data were subjected to analysis of variance. Seeds extracted from fruits were planted and leaf samples taken from young seedlings. The seedling samples were grouped in 10′s and tested for AMV and CMV by ELISA. Percentage infection was calculated using the maximum likelihood estimator formula of Gibbs and Gower [71].

### Detecting virus infection in native plant populations

**Figure 1:**
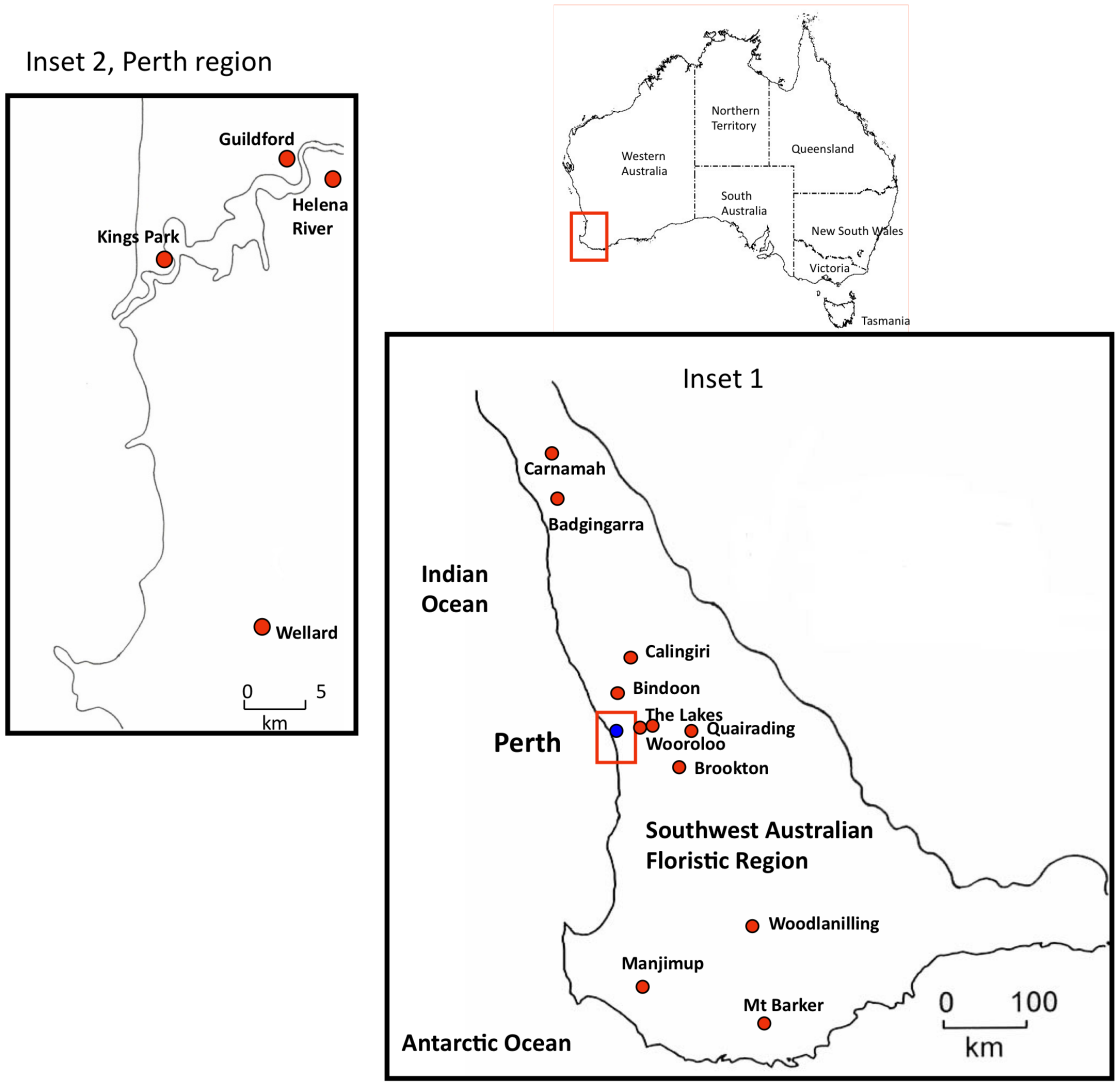
South west Australian floristic region: sites where introduced generalist viruses were found infecting native plants. Insets 1 and 2 show where natural infection with introduced generalist viruses was detected in 2001 and 2009. Inset 1 shows the overall sampling area and Inset 2 shows the urban Perth area. The red spot symbols and place names indicate positions of individual sampling sites with positive virus detections. For an explanation of which viruses were found at each site see Table 5.

Between August and October 2001, flowering shoots of native plants were sampled at diverse sites in the >300 mm rainfall zone in the southwest Australian grainbelt region (Fig. 1, Inset 1). Most sites sampled were natural vegetation stands along roadsides adjacent to pastures or arable crops. The position of each site was recorded using a global positioning system (GPS). Between July and October 2009, flowering shoots of native plants were sampled at sites within the urban Perth area (Fig. 1 Inset 2) or outside it (Fig. 1, Inset 1). Native orchid plants growing within a glasshouse at Kings Park Botanic Gardens were also sampled. The specific locations of the sampling sites were (GPS coordinates in parentheses): Badgingarra (−30.389, +115.501), Bindoon (−31.385, +116.096), Brookton (−32.369, +117.000), Calingiri (−31.092, +116.449), Carnamah (−29.688, +115.887), Guildford (−31.901, +115.977), Helena River (−31.911, +116.045), Kings Park Botanic Gardens (−31.970, +115.822), Manjimup (−34.242, +116.146), Mt Barker (−34.628, +117.662), Quairading (−32.010, +117.401), The Lakes (−31.876, +116.321), Woodanilling (−33.565, +117.431), Wooroloo (−31.802, +116.314), and Wellard (−32.661, +115.835). At each site, the native plant species present were sampled individually (1 shoot/plant). The samples were sealed in polyethylene bags, transported to the laboratory in cooler boxes and stored at 4°C. Prior to testing, the flowering specimens collected were identified and, whenever needed, the Western Australian Herbarium helped with identification. When available, flower tissue was always tested instead of leaf tissue to avoid leaf sap components from native plants that might inhibit the ELISA reaction [72]. In both years, samples were either tested individually or grouped (in 5′s–10′s) before testing. When sufficient grouped samples were present, percentage infection was calculated using the formula of Gibbs and Gower [71].

In 2001, the samples were each tested by ELISA using generic luteovirus, potyvirus and tospoviruses antibodies, and polyclonal *Beet western yellows virus* (BWYV) antibodies. Samples in which potyvirus antigen was detected were retested with BYMV polyclonal antibodies. In 2009, most samples were tested by ELISA using generic potyvirus and tospoviruses antibodies, and some were also tested using polyclonal antibodies to AMV, BYMV, BWYV or CMV. In some instances where infection with BYMV was suspected, only BYMV polyclonal antibodies were used. In one instance where a tospovirus was detected in 2009, the positive sample was retested using polyclonal antibodies to TSWV and *Impatiens necrotic spot virus* (INSV). When positive detections were obtained with BWYV antibodies, these were assumed to have detected TuYV as this virus occurs in native plants in the region and is detected by BWYV antibodies [49], [63], [73], while BWYV *sensu stricto* is not known to occur in Australia.

## Results

### Overall responses to inoculation

Within each individual virus-native plant species combination, the possible responses to inoculation were systemic invasion, localised infection in inoculated leaves only or failure to establish infection, and all three were found. However, presence of localised infection could not be revealed when aphid or graft inoculation were used. When 15 native plant species belonging to eight families were inoculated with 3–12 viruses each (Table 3), 11 species developed systemic infection with 1–5 viruses each, and only localised infection occurred in three species. No infection was detected in one species (*A. flavidus*), despite its inoculation with 11 different viruses. One virus failed to infect any of 11 species inoculated with it, and two viruses failed to infect the single species they were inoculated to. All 14 other viruses established infection in at least one species, all but one of them causing systemic invasion in at least one species.

**Table 3:** Establishment of systemic or localised infection, or failure to establish infection, in 15 native plant species inoculated with introduced and indigenous viruses.

Species	Common name	Viruses causing systemic invasion		Viruses detected only in inoculated leaves		Viruses not detected	
		(No. of viruses detected/no. inoculated)		(No. of viruses detected/no. inoculated)		(No. of viruses not detected/no. inoculated)	
		Introduced	Indigenous	Introduced	Indigenous	Introduced	Indigenous
**Araliaceae**							
*Trachymene*	Blue lace	4/7	0/4	0/7	0/4	3/7	4/4
*coerulea*	flower	(AMV, CMV-SN, PVX, TuMV)				(BYMV-MI, BYMV-FB1, PSbMV, TSWV-Crb)	(ClCV, HarMV-SP1, PaVY-CarP1, PWV)
**Asteraceae**							
*Sonchus*	Native	0/8	0/4	1/8	0/4	7/8	4/4
*hydrophilus*	sowthistle			(CMV-LW, CMV-SN)		(AMV, BYMV-MI, PSbMV, PVX, TSWV-LT, TuMV, TuYV)	(ClCV, HarMV-SP1, PaVY-CarP1, PWV)
**Dilleniaceae**							
*Hibbertia*	Cutleaf	1/3	0/0	0/3	0/0	2/3	0/0
*cuneiformis*	hibbertia	(CMV-LW, CMV-SN)				(BYMV-FB1, TSWV-Crb1)	
**Haemodoraceae**							
*Anigozanthos*	Tall	0/8	0/3	0/8	0/3	8/8	3/3
*flavidus*	kangaroo paw					(AMV, BYMV-MI, CMV-SN, PSbMV, PVX, TSWV-Crb1, TSWV-LT, TuMV, TuYV)	ClCV, HarMV-SP1, PWV)
*Anigozanthos*	Mangles	3/8	0/4	0/8	0/4	5/8	4/4
*manglesii*	kangaroo paw	(CMV-SN, PVX, TSWV-Crb1)				(AMV, BYMV-MI, PSbMV, TuMV, TuYV)	(ClCV, HarMV-SP1, PaVY- CarP1, PWV)
**Fabacaeae**							
*Chorizema*	Holly flame	0/3	0/3	1/3	0/3	2/3	3/3
*ilicifolium*	pea			(CMV-SN)		(PVX, TSWV-Crb1)	(ClCV, HarMV-SP1, PWV)
*Gastrolobium*	Heart leaf	1/8	4/4	1/8	0/4	6/8	0/4
*bilobum*	poison	(BYMV-FB1, BYMV-MI)	(ClCV, HarMV-SP1, PaVY-CarP1, PWV)	(TuMV)		(AMV, CMV-LW, CMV-SN, TSWV-Crb1, PSbMV, PVX,TuYV)	
*Gompholobium*	Hairy	1/3	0/2	0/3	0/2	2/3	2/2
*tomentosum*	yellow pea	(AMV)				(CMV-SN, PVX)	(HarMV-SP1, PWV)
*Hardenbergia*	Native	0/8	1/4	0/8	0/4	8/8	3/4
*comptoniana*	wisteria		(HarMV-SP1)			(AMV, BYMV-FB1, BYMV-MI, CMV-LW, CMV-SN, PSbMV, PVX, TSWV-Crb1, TSWV-LT, TuMV, TuYV)	(ClCV, PaVY-CarP1, PaVY- KnxP1, PaVY-KnxP5, PWV)
*Kennedia*	Coral vine	1/8	4/4	5/8	0/4	3/8	0/4
*coccinea*		(BYMV-MI)	(ClCV, HarMV-Cgt, HarMV-SP1; PaVY-CarP1, PWV)	(AMV, BYMV- FB1, CMV-LW, CMV-SN, PVX, TSWV-Crb1)		(PSbMV, TuMV, TuYV)	
*Kennedia*	Scarlet	3/8	3/4	0/8	0/4	5/8	1/4
*prostrata*	runner	(AMV, CMV-LW, CMV-SN, TuYV)	(ClCV, HarMV-SP1, PWV)			(BYMV-FB1, BYMV-MI, PSbMV, PVX, TSWV-Crb1, TuMV)	(PaVY-CarP1)
**Malvaceae**							
*Alyogyne*	Lilac	0/8	0/4	3/8	1/4	5/8	3/4
*huegelii*	hibiscus			(AMV, CMV-SN, TSWV-Crb1)	(HarMV-SP1)	(BYMV-MI, PSbMV, PVX, TuMV, TuYV)	(ClCV, PaVY-CarP1, PWV)
*Thomasia*	-	1/7	0/4	1/7	2/4	5/7	2/4
*triphylla*		(CMV-SN)		(PVX)	(HarMV-SP1, PWV)	(AMV, BYMV-MI, PSbMV, TSWV-Crb1,TuMV)	(ClCV, PaVY-CarP1)
**Poaceae**							
*Austrostipa*	-	2/4	0/0	1/4	0/0	2/4	0/0
*compressa*		(BSMV, WSMV-Mer1)		(WSMV-Gin)		(BYDV, RyMV)	
**Solanaceae**							
*Solanum*	Native	5/9	0/3	1/9	0/3	3/9	3/3
*symonii*	tomato	(AMV, CMV-SN, PVX, TSWV-Crb1, TuYV)		(PVS)		(BYMV-MI, PSbMV, TuMV)	ClCV, HarMV-SP1, PWV)

For an explanation of virus acronyms and isolates see Table 1. Sap inoculations used, except aphid inoculation used with BYDV and TuYV, and sap, aphid and graft inoculation all used to inoculate *H. comptoniana* with HarMV. Grafting also used to inoculate AMV, BYMV-MI, BYMV-FB1, CMV-SN, CMV-LW, PaVY-KnxP-1, PaVY- KnxP-1, PSbMV and PVX to *H. comptoniana*. Virus detection in leaf samples from inoculated or non-inoculated leaves was by ELISA. Samples from inoculated or tip leaves were grouped separately initially for each virus-plant species combination, but if virus infection was detected each sample was tested individually.

When six introduced generalist viruses were inoculated to 14 native plant species (2-6 viruses/species) belonging to seven different families, 13 species became infected (1–5 viruses each) with infection being restricted to inoculated leaves in three of them (Table 3). CMV infected species belonging to all seven families inoculated including a species in a monocot family (Haemodoraceae), while AMV and TSWV infected species in 4 of 6 and 4 of 7 families, respectively. The corresponding figures for the other three introduced generalist viruses were BYMV (1/7), TuMV (2/6) and TuYV (2/5). Most of these infections involved systemic invasion but numbers of families with infection in which native species only became infected in inoculated leaves were: CMV (1/7), AMV (1/4), TSWV (2/4), BYMV (0/1) and TuMV (1/2) (not applicable to aphid-inoculated TuYV). *S. symonii* and *K. coccinea* became infected with the greatest numbers of introduced generalist viruses, five and four each, respectively. When two different isolates of the same generalist virus were used to inoculate the same native species in five (BYMV), six (CMV) and two (TSWV) instances, respectively, the same infection results were always obtained with both isolates. The one exception was with BYMV where one isolate invaded *K. coccinea* systemically while the other remained restricted to its inoculated leaves.

When two introduced specialist viruses (PVX and PSbMV) were inoculated to 11–12 native plant species each (1–4 viruses/species) and five others were inoculated to one species each, PVS only infected inoculated leaves of *S. symonii* and no infection was obtained with BYDV, RyMV and PSbMV (Table 3). With PSbMV, this was despite it being inoculated to 11 species, four of which were legumes (the family it occurs in naturally). PVX infected five species in five different families and was the only specialist virus that infected species within four of these families. The five families it infected included four dicots and the monocot family Haemodoraceae. When the introduced specialist viruses BSMV and WSMV were inoculated to plants of the native grass species *Austrostipa compressa*, BSMV and one isolate of WSMV infected it systemically, but a second isolate of WSMV remained localised within inoculated leaves.

When four indigenous viruses were inoculated to 14 native plant species (2–4 viruses/species), six species became infected with 1–4 viruses each (Table 3). Among the legumes, 4 of 6 species became infected by 1–4 viruses each and these infections were always systemic. All four indigenous viruses infected *K. coccinea* and *Gastrolobium bilobum* (ClCV, HarMV, PaVY, PWV), and three infected *K. prostrata* (ClCV, HarMV, PWV). *H. comptoniana* only became infected by the indigenous virus that infects it naturally (HarMV), despite sap and aphid inoculation with 12 and 11 different viruses, respectively. Also, when it was graft inoculated with eight different viruses, it again only became infected by HarMV. Two malvaceous species became infected by 1–2 of them each (HarMV, PWV), but in inoculated leaves only. When 2–3 isolates of two indigenous viruses were inoculated to plants of two species, both HarMV isolates infected inoculated leaves of *K. coccinea* but none of three PaVY isolates established infection in *H. comptoniana*. None of the four indigenous viruses infected species in 4 of 6 other families.

Overall, there were 16 instances involving eight native species where 2–3 isolates of a virus were inoculated to the same species. Only in two of these 16 instances was there any difference between the way the isolates responded (BYMV in *K. coccinea* and WSMV in *A. compressa*).

### Symptom severity

**Figure 2:**
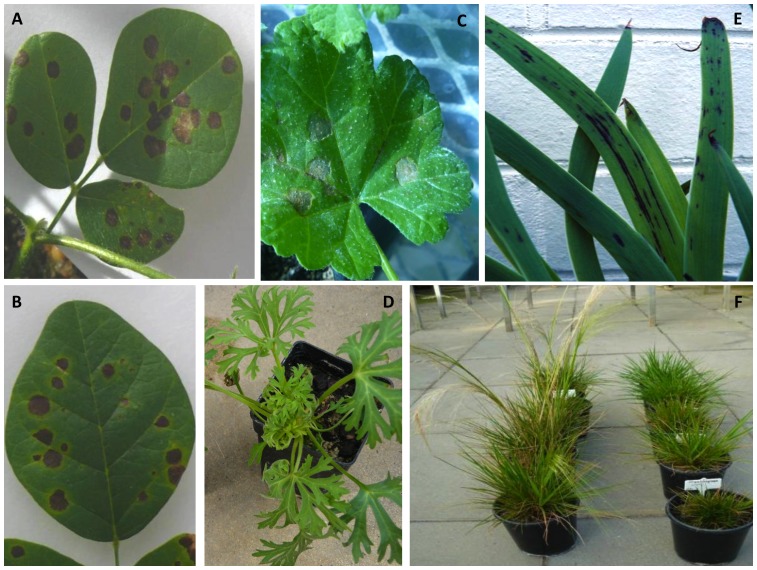
Symptoms in native plant species inoculated with infective leaf sap containing introduced or indigenous viruses. A, Large necrotic spot lesions caused by localised infection with an indigenous virus (Clitoria chlorosis virus) in an inoculated leaf of *Kennedia coccinea*; B, Large necrotic spot local lesions caused by localised infection with an introduced specialist virus (*Potato virus X*, PVX) in an inoculated leaf of *K. coccinea*; C, Large necrotic spot local lesions caused by localised infection with an introduced generalist virus (*Tomato spotted wilt virus*) in an inoculated leaf of *Alyogyne huegelii*; D, Mild bunching symptoms in young leaves in a plant of *Trachymene coerulea* caused by systemic infection with an introduced generalist virus (*Alfalfa mosaic virus*); E, Mild symptoms of necrotic streaking and necrotic leaf markings caused by systemic infection with an introduced specialist virus (PVX) in a plant of *Anigozanthos manglesii*; F, Mild-moderate stunting caused by systemic infection with an introduced specialist virus (*Wheat streak mosaic virus*) in plants of *Austrostipa compressa* (right) compared with more vigorous growth in a mock-inoculated plants (left).

When viruses infected inoculated leaves of native plant species, severe necrotic spot or ring lesions sometimes developed either associated with systemic symptoms (Table 4; Fig. 2), e.g. in *K. coccinea* with ClCV (Fig. 2A), or where there was no systemic invasion, e.g. in *K. coccinea* with PVX (Fig. 2B) and *Alyogyne huegelii* with TSWV (Fig. 2C). This occurred with generalist or specialist introduced viruses and indigenous viruses. In other instances, inoculated leaves developed mild symptoms, such as chlorotic spots or rings, and this also occurred with all three categories of virus. Inoculated leaves developed symptomless infection in four species lacking systemic infection: *Chorizema ilicifolium* and *Sonchus hydrophilus* (CMV), *A. huegelii* (AMV, HarMV) and *A. compressa* (WSMV-Gin), and in several others where systemic invasion occurred, e.g. in *Trachymene coerulea* (AMV, TuMV).

**Table 4:** Responses of native plants infected following inoculation with introduced and indigenous viruses.

Species	Common name	Virus	No. of plants	Symptoms	
		(Isolate code included, if >1 isolate used)	Infected/no. inoculated	Inoculated leaves	Non-inoculated leaves
**Araliaceae**					
*Trachymene coerulea*	Blue lace flower	AMV (G)	4/10	SI	DC, LBU, St
		CMV-SN (G)	14/15	LCS, SI	MM, C, DC
		PVX (S)	11/15	LNS, SI	MM
		TuMV (G)	7/15	SI	M, C, DC, LD, NSST, St, SN, PD
**Asteraceae**					
*Sonchus hydrophilus*	Native sowthistle	CMV-LW (G)	3/5	SI	NI
		CMV-SN (G)	6/11	SI	NI
**Dilleniaceae**					
*Hibbertia cuneiformis*	Cutleaf hibbertia	CMV-LW (G)	2/5	LNR	MM, SCR
		CMV-SN (G)	1/5	LNR	SS
**Haemodoraceae**					
*Anigozanthos manglesii*	Mangles kangaroo paw	CMV-SN (G)	4/16	CLST, NLST	MM, C, CLST, NLST, St
		PVX (S)	8/9	CLST, NLST	CLST, NLST
		TSWV-Crb1 (G)	3/5	CLST, NLST	SCS, SNS, NLST, C, SN, St, PD
***Fabaceae***					
*Chorizema ilicifolium*	Holly flame pea	CMV-SN (G)	1/5	SI	NI
*Gastrolobium bilobum*	Heart leaf poison	ClCV (I))	4/5	ELNS	M
		BYMV-FB1 (G)	3/5	ELCS	SS
		BYMV-MI (G)	5/5	SI	MM, DC, LD, SHS, EN, St
		HarMV-SP1 (I)	9/10	ELCS, ELNS, SI	M, SCS, C, STD, St
		PaVY-CarP1 (I)	2/10	SI	MM, CLP
		PWV (I)	14/20	ELNS, SI	VC, M, LD, SHS, EN, St
		TuMV (G)	9/11	ELNS	NI
*Gompholobium tomentosum*	Hairy yellow pea	AMV	1/6	SI	SN, PD
*Hardenbergia comptoniana*	Native Wisteria	HarMV-SP1 (I)	1/5	SI	M
		HarMV-SP1* (I)	0/5	-	-
		HarMV-SP1** (I)	4/5	N/A	VC, SCS, M, LD
*Kennedia coccinea*	Coral vine	AMV (G)	12/13	ELCS, LNS, ELNS	NI
		BYMV-FB1	2/5	LCR, NLP, SI	NI
		BYMV-MI (G)	7/10	LCS, ELCS	VC, SCR, M, C, LD
		ClCV (I)	5/5	LNS, ELNS, VN	VC, M, VB, C, SNR, SNF, VN, DC, LD, STD, SN, St
		CMV-LW (G)	4/5	LCR, LNS, ELNS	NI
		CMV-SN (G)	9/15	LCS, LNS, ELNS	NI
		HarMV-Cgt	1/3	ELNS	VC, M, C
		HarMV-SP1 (I)	6/8	ELCS, ELNS	VC, M, C, LD, SNS, STD, SN
		PaVY-CarP1 (I)	1/5	ELCS, LNS, ELNS, VN	NI
		PVX (S)	10/10	LNS, ELNS	NI
		PWV (I)	6/7	LNS, ELNS, VN	VC, SCS, M, C, SNF, LD, VN, St, STD, SN
		TSWV-Crb1	8/12	LCR, LCS, ELCS, ELNS	NI
*Kennedia prostrata*	Scarlet runner	AMV (G)	10/15	LNS, SI	MM,VN, STD, St
		ClCV (I)	8/10*	ELCS, ELNS, VN	M, LD, VN, STD, SN, St, PD
		CMV-LW (G)	3/5	SI	VC, MM, MLN, LD, St
		CMV-SN (G)	7/10	ECLS, ELNS	STD, SN, PD
		HarMV-SP1 (I)	3/5	ELCS, ELNS	VC, M, LD, STD, St
		PWV (I)	4/10	ELCS, ELNS	VC, M, St, PD
		TuYV* (G)	2/5	N/A	SS
**Malvaceae**					
*Alyogyne huegelii*	Lilac hibiscus	AMV (G)	5/5	SI	NI
		CMV-SN (G)	5/10	LCS	NI
		HarMV-SP1 (I)	3/10	SI	NI
		TSWV-Crb1 (G)	9/10	LCS, LCR, ELCR, ELNS	NI
*Thomasia triphylla*	-	CMV-SN (G)	5/5	LCS, LCR, LNS, ELNS	SS
		HarMV-SP1 (I)	4/5	LCS, LCR, ELNS	NI
		PVX (S)	4/5	ELNS	NI
		PWV (I)	1/5	LCS	NI
**Poaceae**					
*Austrostipa compressa*	-	BSMV ES-1 (S)	5/5	SI	SS
		WSMV-Gin (S)	2/5	SI	NI
		WSMV-Mer1 (S)	7/10	CLST, SI	CLST, St
**Solanaceae**					
*Solanum symonii*	Native tomato	AMV-EW (G)	5/5	SI	M, LD, PLD
		CMV-SN (G)	5/5	SI	M, LD
		PVS (S)	8/10	LCR, SI	NI
		PVX (S)	5/5	SI	SCS
		TSWV-Crb1 (G)	10/10	LNS, LNR, ELNR	SCS, SCR, MM, LD, TLBU, STD, St
		TuYV* (G)	5/5	N/A	LLBR, PLD

For an explanation of virus acronyms and isolates see Table 1. G =  Generalist, S =  Specialist, I =  Indigenous. Sap inoculation used except with HarMV to *H. comptoniana* (sap, aphid and graft inoculation all used), and TuYV (only aphid inoculation used), * =  aphid inoculation, ** =  graft inoculation. Virus detection in leaf samples from inoculated or non-inoculated leaves was by ELISA. Samples from inoculated or tip leaves were grouped separately initially for each virus-plant species combination, but if virus infection was detected each sample was tested individually.

Coded symptom descriptions:

Inoculated leaves – CLP, chlorotic line patterns; CLST, chlorotic leaf streaking; ELCR, expanding local chlorotic rings; ELCS, expanding local chlorotic spots or blotches; ELNR, expanding local necrotic rings; ELNS, expanding local necrotic spots; LCR, local chlorotic rings; LCS, local chlorotic spots or blotches; N/A  =  Not applicable; NLP, necrotic line patterns; LNR, local necrotic rings; LNS, local necrotic spots; NLST, necrotic leaf streaking; SI, symptomless infection; VN, veinal necrosis.

Non-inoculated leaves – C, chlorosis or palor; CLP, chlorotic line patterns; CLST, chlorotic leaf streaking; DC, downcurling of leaves; EN, enations; LBU, leaf bunching; LD, leaf deformation; LLBR, lower leaf bronzing; LNS, systemic necrotic spotting; M, mosaic; MM, mild mosaic; NI, not infected; NLST, necrotic leaf streaking; NSST, necrotic stem streaking; PD, plant death; PLD, premature leaf drop; SCR, systemic chlorotic rings; SCS, systemic chlorotic spots or blotches; SHS, shoestring symptoms; SN, systemic necrosis; SNF, systemic necrotic flecking; SNR, systemic necrotic rings; SS, symptomless systemic infection; SC, St, stunting; STD, shoot tip death; TLBU, tip leaf bunching; VB, vein banding; VC, vein clearing; VN, veinal necrosis.

Symptomless systemic infection developed with four combinations of plant species and introduced generalist viruses (*G. bilobum*, BYMV-FB1; *Hibbertia cuneiformis*, CMV-SN; *K. prostrata*, TuYV; *Thomasia triphylla*, CMV-SN;) and one combination involving an introduced specialist virus (*A. compressa*, BSMV) (Table 4). It did not occur when indigenous viruses invaded plants systemically.

Mild systemic symptoms, such as mottle, systemic chlorotic spotting, deformation and necrotic streaking of leaves, developed in nine species with 10, four and four combinations of native plant species infected with generalist, specialist or indigenous viruses, respectively (Table 4; Fig. 2). Four introduced generalist viruses induced such symptoms in at least one of six species belonging to four families, *H. cuneiformis* (CMV-LW), *S. symonii* (AMV, CMV-SN, TuYV) and *T. coerulea* (AMV, CMV-SN) (Fig 2D), *G. bilobum* (BYMV-MI), *K. coccinea* (BYMV-MI), *K. prostrata* (AMV, CMV-LW). Two introduced specialist viruses induced them in four families, *A. manglesii* (PVX) (Fig. 2E), *S. symonii* (PVX), *A. compressa* (WSMV-Mer1) (Fig. 2F), and *T. coerulea* (PVX). They were also induced by four indigenous viruses in two species belonging to one family, *G. bilobum* (ClCV, PaVY, PWV) and *H. comptoniana* (HarMV).

**Figure 3:**
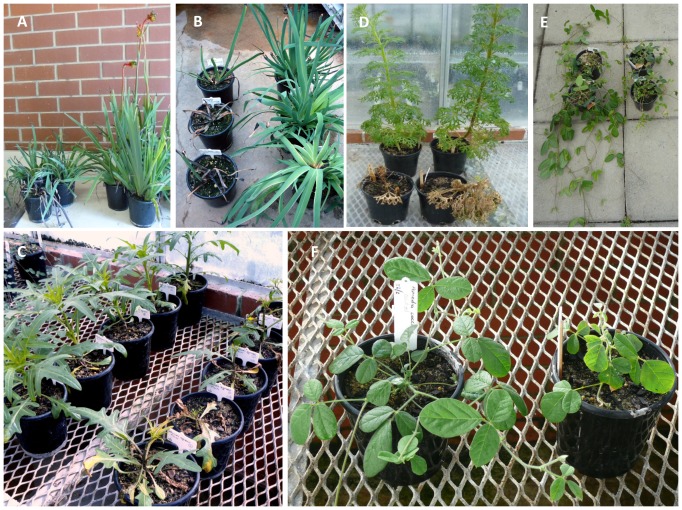
Severe symptoms in native plant species inoculated with infective sap containing introduced or indigenous viruses. A, Severe stunting caused by systemic infection with an introduced generalist virus (*Cucumber mosaic virus*) in two plants of *Anigozanthos manglesii* (left) compared with healthy vigorous growth in two mock-inoculated plants of *A. manglesii* (right); B, Severe plant stunting, apical shoot necrosis and leaf necrosis caused by recent infection with an introduced generalist virus (*Tomato spotted wilt virus*, TSWV) in three plants *A. manglesii* (left) compared with healthy vigorous growth in three recently mock-inoculated plants of *A. manglesii* (right); C, Severe plant stunting, leaf necrosis and leaf chlorosis caused by infection with TSWV in five plants of *Solanum symonii* (right) compared with healthy vigorous growth in five mock-inoculated plants of *S. symoniii* (left); D, Systemic necrosis and death caused by infection with an introduced generalist virus (*Turnip mosaic virus*) in two plants of *Trachymene coerulea* (front) compared with healthy vigorous growth in two mock-inoculated *T. coerulea* plants (back); E, Severe stunting caused by systemic infection with an indigenous virus (Clitoria chlorosis virus) in two plants of *Kennedia coccinea* (right) compared with healthy vigorous growth in two mock-inoculated plants of *K. coccinea* (left). F, Severe stunting caused by recent systemic infection with an indigenous virus (*Passion fruit woodiness virus*) in a plant of *K. coccinea* (right) compared with healthy vigorous growth in a recently mock-inoculated plant of *K. coccinea* (left).

Severe systemic symptoms that included severe stunting, apical necrosis or plant death developed in seven of the 15 native plant species inoculated (Table 4; Fig 3). Four introduced generalist viruses induced such symptoms in six combinations of introduced virus and native plant species. The six combinations involved five species belonging to four families (1-2 viruses/species): *A. manglesii* (CMV-SN, TSWV Crb-1) (Figs 3A, 3B), *Gompholobium tomentosum* (AMV), *K. prostrata* (CMV-SN), *S. symonii* (TSWV Crb-1) (Fig. 3C), and *T. coerulea* (TuMV) (Fig. 3D). Three indigenous viruses induced them in seven combinations of virus and plant species (1–3 viruses/species). The seven combinations involved three species belonging to one family: *G. bilobum* (HarMV), *K. coccinea* (ClCV, HarMV, PWV) (Fig. 3E, F), and *K. prostrata* (ClCV, HarMV, PWV). None of the introduced specialist viruses induced severe systemic symptoms.

### Effects on plant growth and biomass

To quantify the effects of virus infection on the growth of native plants, small-scale, paired healthy and virus-infected plant comparisons were made with nine virus-host combinations. Where severe systemic symptoms developed, foliage dry weight was always significantly diminished by virus infection (*P*<0.05). Three of these five virus-host combinations involved indigenous viruses and two involved introduced generalists. The losses in biomass recorded were 87% for *K. coccinea* with ClCV, 97% for *K. prostrata* with ClCV, 96% for *G. bilobum* with HarMV, 53% for *A. manglesii* with CMV and 100% for *T. coerulea* with TuMV. Thus, both indigenous and introduced generalist viruses caused large foliar biomass losses. When similar paired comparisons were made for four host-virus combinations where mild systemic symptoms developed, no significant biomass decreases were recorded. These combinations were *T. coerulea* with PVX, CMV and AMV, and *A. manglesii* with PVX.

In a long duration experiment in which the effects of systemic infection with TSWV, CMV and mixed infection with AMV and CMV were compared in plants of *S. symonii*, TSWV caused very severe symptoms, AMV + CMV caused moderately severe symptoms and CMV alone caused mild symptoms. TSWV alone or AMV + CMV both caused significant losses in foliage dry weight (*P*<0.001), but CMV alone did not (Table S1). The foliage dry weight losses induced by TSWV alone or AMV + CMV were 94% and 51%, respectively. The mean reductions in total fruit biomass arising from virus infection were not significantly different from those of the healthy control plants. However, the mean individual fruit dry weight values were significantly different (*P* = 0004): the reductions caused by virus infection were 100% (TSWV), 47% (AMV + CMV) and 40% (CMV). Seeds from plants infected with CMV or AMV + CMV were smaller sized with less regular shapes than seeds from healthy control plants. When leaf samples from 1,010 seedlings grown from seeds from plants infected with AMV + CMV or CMV alone were tested, a CMV seed transmission rate to seedlings of 0.4% was detected. No CMV or AMV were detected in seedlings grown from 710 seeds from healthy control plants, or AMV in seedlings grown from 500 seeds from plants infected with AMV + CMV.

In a second similar experiment of shorter duration with *S. symonii*, infection with TSWV and AMV caused very severe and mild systemic symptoms respectively, and both viruses significantly diminished foliage dry weights (*P*<0.001) (Table S1). The foliage dry weight reductions recorded were 71% (TSWV) and 34% (AMV).

### Natural infections detected

**Figure 4:**
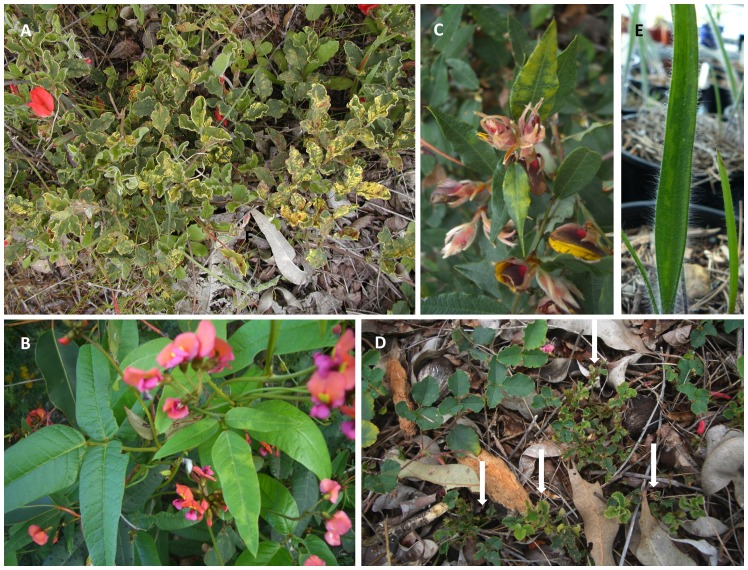
Native plants growing in the South West Australian Floristic Region showing obvious virus symptoms. For locations of site names see Fig. 1. A, Severe chlorotic mottle and leaf deformation caused by infection with an introduced generalist virus (*Bean yellow mosaic* virus, BYMV) in plants of *Kennedia prostrata* growing near Badgingarra; B, Mild mosaic caused by infection with an unidentified virus in young leaflets (right) of *K. coccinea* plants growing near Wooroloo; C, Bright yellow leaf mottle caused by infection with an unidentified virus in young leaves of a *Bossiaea sp*. plant growing at The Lakes; D, Chlorotic leaf mottle, leaf deformation and severe plant stunting (plants with arrows) caused by infection with an unidentified virus in plants of *K. prostrata* growing near Wooroloo compared with vigorously growing healthy *K. prostrata* plants (top left and right); E, Leaf mosaic caused by infection with BYMV in leaf of *Caladenia paludosa* growing in an orchid collection at Kings Park.

Fig. 4 shows examples of typical symptoms caused by natural infection with BYMV (Fig. 4A) or unidentified viruses (Fig. 4B–D) in native plants growing at the agro-ecological interface in the SWAFR or by BYMV in a plant growing in a native orchid collection (Fig. 4E).

In 2001, virus infection was detected by ELISA in samples from 10 native plant species belonging to three families growing at nine sites in non-urban or agricultural zones of the SWAFR. These sites were within an overall sampling area of the grainbelt region measuring >600 Km north to south (Fig. 1, Inset 1; Table 5). Potyvirus infection was found in five legume species (number of sites positive/total number of collection sites in parentheses), *Gompholobium* spp. (1/8), *H. elliptica* (1/2), *K. eximia* (1/3), *K. prostrata* (1/9), and *Leptosema aphyllum* (1/2), and in one species of Caesalpiniaceae, *Cassia* sp. (1/2). Retesting of potyvirus positive samples detected BYMV in *K. eximia*, *K. prostrata* and *Cassia* sp. A distinct leaf mottle was present in BYMV-infected *K. eximia*. BWYV antibodies detected TuYV in two legume species, *B. ornata* (1/2) and *Daviesia nudiflora* (1/2), and one species of Goodeniaceae, *Damperia* sp. (1/9). The single generic luteovirus detection corresponded to the BWYV antibody positive *Damperia* sp. sample, but the other TuYV positive samples gave negative results when tested with generic luteovirus monoclonal antibody. Tospovirus infection was detected in one sample of legume species *B. eriocarpa* (1/11). When infection incidences were examined within randomly collected samples at five infected sites, those found were: *L. aphyllum* (14% potyvirus), *K. eximia* (13% BYMV), *Damperia* sp. (10% TuYV), *B. eriocarpa* (3% TuYV) and *Bossiaea eriocarpa* (1% tospovirus). Overall, introduced generalist viruses were identified at six of the nine sites with virus infection, three each with BYMV or TuYV.

**Table 5:** Native plants in which infection with introduced or unidentified viruses was detected by ELISA tests on samples.

Species	Common name	Site location	No. of plants tested (grouping)	No. of positive samples (% infection)						
				AMV	TuYV*	BYMV	CMV	Potyvirus	Tospovirus	Luteovirus
2001 Samples										
Caesalpiniaceae										
*Cassia* sp.	-	Calingiri	1 (1)	-	0	1	-	1	0	0
Fabaceae										
*Bossiaea eriocarpa*	Common brown pea	Bindoon	85 (5)	-	0	-	-	0	1 (1)	-
*Bossiaea ornata*	Broad-leaf brown pea	The Lakes	5 (1)	-	1	-	-	0	0	-
*Daviesia nudiflora*	-	Quairading	40 (1)	-	1 (3)	-	-	0	0	0
*Gompholobium* sp.	-	Badgingarra	7 (7)	-	0	-	-	1	0	-
*Hovea elliptica*	Tree hovea	Mt Barker	10 (10)	-	0	-	-	1	0	0
*Kennedia eximia*	-	Bindoon	30 (5)	-	0	3 (13)	-	3 (13)	0	0
*Kennedia prostrata*	Scarlet runner	Brookton	15 (5)	-	0	2	-	2	0	0
*Leptosema aphyllum*	Ribbon pea	Carnamah	21 (7)	-	0	-	-	2 (14)	0	0
Goodeniaceae										
*Damperia* sp.	-	Woodanilling	10 (1)	-	1 (10)	-	-	0	0	1 (10)
2009 Samples										
Asparagaceae										
*Chamaescilla corymbosa*	Blue squill	Kings Park	12 (1)	0	0	-	0	12 (100)	0	-
**Droseraceae**										
*Drosera* sp.	Sundew	Wooroloo	6 (6)	-	0	1	0	1	0	-
Fabaceae										
*Hovea elliptica*		Wellard	1	-	-	-	0	0	1**	-
**Haemodoraceae**										
*Anigozanthos* sp.	Kangaroo paw	Manjumup	60 (10)	0	0	-	0	3 (8)	0	-
*Anigozanthos manglesii*	Mangles kangaroo paw	Wooroloo	1	-	-	-	1	0	0	-
**Hemerocallidaceae**										
*Caesia micrantha*	Grass lily	Kings Park	30 (1)	-	-	-	-	14 (47)	-	-
Juncaginaceae										
*Triglochlin sp.*	Arrowgrass	Helena River	20 (1)	-	-	11(55)	-	-	-	-
*Triglochlin sp.*	Arrowgrass	Guildford	20 (1)	-	-	13 (65)	-	-	-	-
*Triglochlin sp.*	Arrowgrass	Kings Park	18 (1)			18 (100)	-	18 (100)		
*Triglochlin sp.*	Arrowgrass	Not recorded	50 (1)	-	0	-	-	47 (94)		
Orchidaceae										
*Caladenia paludosa*	Common swamp spider-orchid	Kings Park^+^	2	-	-	2	0	2	0	-
*Cymbidium canaliculatum*	Black orchid	Kings Park^+^	1	-	-	0	0	1	0	-
*Dendrobium* sp.	-	Kings Park^+^	1	-	-	0	0	1	0	-
*Diuris longifolia*	Common donkey orchid	Kings Park^+^	3 (1)	-	-	2	0	3	0	-
*Diuris longifolia*	Common donkey orchid	Kings Park^+^	46 (1)	-	-	12 (26)	0	25 (54)	0	-
*Diuris micrantha*	Dwarf bee orchid	Kings Park^+^	1	-	-	1	0	1	0	-
*Microtis sp.*	Onion orchid	Kings Park^+^	1	-	-	1	0	1	0	-
*Thelymitra sp.*	Sun orchid	Kings Park^+^	1	-	-	1	0	1	0	-

For an explanation of virus acronyms see Table 1, - =  Not tested, *  =  TuYV detected by BWYV polyclonal antibodies. **  =  Tospovirus positive sample tested negative for TSWV and *Impatiens necrotic spot virus* (INSV), +  =  Native orchid collection. Samples were either tested individually or grouped (in 5′s–10′s) before testing. When sufficient grouped samples were present, percentage infection was calculated using the formula of Gibbs and Gower [71]. All orchid samples also tested for *Cymbidium mosaic virus* and *Odontoglossum ringspot virus* by ELISA, but none contained them.

In 2009, virus infection was detected by ELISA in samples from eight native plant species at four sites within the urban Perth area (Table 5; Fig. 1, Inset 2) and two further afield, including Manjimup >300 Km south of Perth (Table 5; Fig. 1, Inset 1). Potyvirus infection was found in one species each of (numbers of sites positive/total number of collection sites): Asparagaceae, *Chamaescilla corymbosa* (1/1); Droseraceae, *Drosera* spp. (1/3); Haemodoraceae, *Anigozanthos* sp. (1/5); Hemerocallidaceae, *Caesia micrantha* (1/1); and Juncaginaceae, *Triglochlin sp*. (2/2). Retesting of potyvirus positive samples detected BYMV only in *Drosera* sp. and *Triglochlin* sp., but testing of additional *Triglochlin* sp. samples from two other sites also detected this virus. Tospovirus infection was detected in one sample of legume species *H. elliptica* (1/3), but retesting the positive sample with TSWV and INSV antibodies failed to detect either virus. CMV was detected in one species of Haemodoraceae, *A. manglesii* (1/2) at the site furthest from the urban Perth area (Manjimup). When incidences of infection were examined within randomly collected samples of species at infected sites, potyvirus incidences found were: *C. corymbosa* 100%, *Anigozanthos* sp. 8% and *C. micrantha* 47%. The incidences of BYMV in random samples of *Triglochlin* sp. from three sites were 100%, 55% and 65%.

In 2009, when plants of native orchid species growing in a glasshouse containing native orchid plants being propagated prior to reintroduction to the wild were inspected for virus symptoms, several showed leaf mosaic and plant stunting (Fig. 4E). When leaf samples from these orchid plants were tested, both potyvirus and BYMV infection were found in plants of *Caladinia paludosa*, *Microtis* sp., *Thelymitra* sp., *Diuris longifolia* and *D. micrantha* (Table 5). Potyvirus infection without BYMV was detected in other plants of *D. longifolia*, and in plants of *Cymbidium canaliculatum* and *Dendrobium* spp. The incidences of potyvirus and BYMV infection in random samples from plants of *D. longifolia* were 54% (potyvirus) and 26% (BYMV). No tospovirus or CMV infection was detected in any native orchid samples.

## Discussion

This study provides evidence supporting our three original hypotheses. We found (i) the introduced generalist and indigenous viruses both caused severe systemic symptoms and growth reductions when they infected some native plant species, (ii) the specialist viruses caused only mild or symptomless systemic infection, and (iii) three introduced generalist viruses were detected in natural vegetation at sites distributed widely at agro-ecological interfaces in the SWAFR. Our research highlights the potential for serious damage to plant biodiversity to occur from virus disease epidemics that arise from new encounters between introduced generalist viruses and native plants. Such new encounters are most likely to occur near the agro-ecological interface between managed and natural ecosystems. The introduced generalist viruses could then proceed to invade undisturbed native plant communities. Some introduced generalist viruses infected plants in more families than others and so pose a greater potential threat. Our research also highlights the potential for serious damage to plant biodiversity from indigenous viruses in disturbed natural vegetation when they encounter hosts they are poorly adapted to. Indeed, the indigenous viruses tested were often surprisingly virulent when they infected native plant species. Although the comparisons made were less comprehensive for the introduced specialist viruses than the generalist and indigenous viruses, the specialist viruses studied seemed less cause for immediate concern.

Fitness is a critical factor when considering wild plant ecology. When virus-infected plants are growing in mixed species communities, relative fitness of infected plants refers to survivorship arising from their abilities to compete with healthy plants of other species, reproduce sufficiently and produce the next generation of seedlings [2], [18], [37]. Because of (i) logistical issues when handling large numbers of viruses and native plant species in the field and (ii) restrictions on deliberately introducing viruses into natural plant communities potentially containing endangered species, we were unable to undertake long-term *in situ* field observations over several generations to examine the effects of virus infection and their effects on biodiversity. Instead, we adopted the simpler, alternative strategy of inoculating viruses to native plant species in the glasshouse, and documenting the consequences of doing this. Bearing in mind that factors such as climate, soil, plant genotype, plant age at time of infection and virus strain can all influence symptom severity in the field and the method of plant inoculation used was mostly artificial (sap inoculation), we are cautious not to over-interpret the significance of the symptom reactions induced by inoculation under glasshouse conditions. Moreover, the waxy leaf surfaces of some native species probably diminished the success of some sap inoculations (e.g., in *A. flavidus* and *H. comptoniana*). Also, we have no information on the feeding behaviour of virus vectors on the native plant species used, including the aphid, thrips or eriophyid mite vectors of the viruses we studied. In addition, our results included data on the likelihood of variation due to virus strain as plants belonging to eight native species responded differently in two out of 16 instances in which 2–3 isolates of the same virus were inoculated to them. The difference in these two instances was that one isolate remained localised to inoculated leaves but the other moved systemically. However, despite such limitations, taken as a whole the data obtained do indicate the potential of each virus to move systemically and cause severe, mild or no systemic symptoms in native plant species growing in the wild.

The extent to which systemic symptom data can be used to indicate diminished survivorship of virus-infected plants growing with healthy ones of other species in mixed native plant species communities needs careful consideration. In communities in which one or more species develop severe systemic symptoms and other species are non-hosts, the competitive and reproductive capacities of the affected species are likely to diminish to a greater extent than where systemic symptoms are mild or absent in infected plants. However, when virus infection is so severe that it causes systemic necrosis that kills individual plants rapidly (Fig. 3D), it prevents them from becoming sources of virus inoculum for spread to other plants and so may be less damaging than where virus-infected plants develop severe symptoms but still survive [74]–[77]. *A. manglesii*, *K. coccinea* and *K. prostrata* are examples of species commonly found growing next to each other naturally in the SWAFR and the predominant reactions of each of them to CMV infection by sap inoculation ranged from systemic necrosis and plant death (*K. prostrata*) and severe stunting (*A. manglesii*), to localised infection only (*K. coccinea*). It would be interesting to determine in *in-situ* studies if aphids transmit CMV to these same plant species causing similar reactions and whether systemic necrosis that kills infected plants largely prevents *K. prostrata* from becoming a source of virus inoculum for spread to other plants. From studies where systemic virus symptoms are mild in a species, such infections still have the capacity to alter the species balance by decreasing the ability of infected plants to compete with healthy plants of non-host species or providing a virus inoculum source for virus spread to more sensitive and vulnerable host species (see Introduction). Thus, although viruses that cause severe systemic symptoms in a given species without killing it are likely to decrease survivorship more than where systemic symptoms are mild, both are likely to (i) reduce competitive ability, reproduction and recruitment, and (ii) alter species composition in natural vegetation.

Our findings from the virus inoculation and biomass loss studies suggest that, when they spread in mixed species populations of host and non-host native plants, both introduced generalist viruses and indigenous viruses have the potential to cause considerable losses in competitive and reproductive capacities of native species that become infected. This is because (i) severe systemic symptoms that included stunting or plant death developed with six or seven combinations of native plant species and introduced generalist or indigenous viruses, respectively, but in none of the combinations of specialist virus and native plant; (ii) major losses in foliage biomass (53-95%) were recorded where severe systemic symptoms developed with different combinations of introduced generalist virus or indigenous virus and native plants; and (iii) where mild systemic symptoms developed statistically significant losses in foliage biomass were recorded in only two instances with *S. symonii* (34–40%) and the viruses involved were both introduced generalists. Studies on losses in reproductive capacity were hampered because the native plant species grown remained vegetative under glasshouse conditions, except with *S. symonii*. In this species, mixed infection with two generalist viruses (AMV and CMV) resulted in increased symptom severity, and losses in foliage and fruit biomass over what occurred when either virus was present alone. No seed was produced by the TSWV-infected plants but seed from plants infected with AMV + CMV or CMV alone was small and misshapen. Moreover, the 0.4% CMV seed transmission rate found in *S. symonii* raises the question of virus carry over from one generation to the next through seeds. Other evidence that introduced generalist viruses can greatly decrease foliage and seed production of Australian native plants comes from study of the impact of AMV on foliage and seed production in the native Australian legume *Cullen australicum* [78]. Thus, mixed virus infection in native plants growing at the interface between managed and natural vegetation is likely to further magnify reductions in competitive and reproductive capacities of infected plants.

The surprisingly severe systemic symptoms induced by indigenous viruses in native plant species (Fig. 3D–F) does not support the suggestion that indigenous viruses are likely to be harmless to them (see Introduction). They indicate instead that they have the potential to cause serious damage when they make host species jumps [79] within populations of disturbed native flora. Moreover, our failure to reproduce BYMV infection in the commonly naturally BYMV-infected host *K. prostrata* may reflect the need for its adaptation to this host after initially invading it. A BYMV isolate from naturally infected *K. prostrata* proved poorly infectious on sap inoculation to *Trifolium subterraneum* plants (R.A.C. Jones unpubl.), although this widely grown species is very frequently BYMV-infected in annual clover pastures in the region [42], [43], [50]. This finding illustrates how new encounters at the agro-ecological interface between managed and native ecosystems in the SWAFR present an ideal opportunity to study virus evolution, pathogenesis, host species jumps, and the rate of adjustment of viruses to survive in completely new hosts [79]–[84].

The six introduced generalist and seven introduced specialist viruses studied infected 13 and six species in seven and six families, respectively. However, among the seven specialist viruses, PVX accounted for four of the six infected families on its own and also infected one of the other two families, while PSbMV failed to infect any of the four legume and seven other native species in five other families it was inoculated to. PVX is classed as a specialist because of its narrow known natural host range [8], [85], but it behaved more like a generalist when artificial inoculation was used as it infected native plants in both dicot and monocot families. Further study of its natural host range might reveal that PVX is actually a generalist. Among the introduced generalist viruses, CMV infected native plant species within all seven families inoculated including a species within a monocot family, AMV and TSWV infected species in four families each, and BYMV, TuMV and TuYV infected species in 1–2 families each. This indicates that in situations where virus reservoirs are of similar magnitude at the interface between managed and natural vegetation, CMV is likely to have the greatest potential to invade a diverse range of native plant species, followed by AMV and TSWV. The four indigenous viruses infected legume species and two of them infected malvaceous species. In previously reported host range studies using sap inoculation to non-native species in the glasshouse, ClCV, PaVY, HarMV and PWV infected species of three, four, three and three additional families, respectively [15], [33].

When pastures or crops grow alongside native plants in the SWAFR's numerous ancient ecosystem - recent agroecosystem interfaces, native plants are likely to encounter 14 of the 17 viruses used in this study. Previously, five introduced viruses were reported naturally infecting native plants in the SWAFR (see Introduction). Here, we provide further examples of three introduced generalist viruses spreading to native plant species in non-urban or agricultural areas at sites covering a wide region (sampling area measured >600 Km north to south): BYMV and TuYV, and the first evidence of CMV invading them. In addition, unidentified potyviruses and tospoviruses were also detected. Some of the unidentified virus infections we found in diverse hosts may represent introduced viruses not detectable by the antibodies used to test samples, but which might have been detected by more sensitive testing procedures, such as RT-PCR or next generation sequencing [49], [86]. Others may have been caused by indigenous viruses, such as the indigenous potyviruses reported in deep sequencing studies [49], [66], [67]. Overall, in this and previous studies BYMV is the introduced virus found infecting the most native plant species at the greatest number of sites. This is not surprising because, as mentioned above, BYMV-infected pastures dominated by the introduced species *T. subterraneum* occur frequently in the region, especially in high rainfall zones [22], [43], [50]. Also, BYMV often infects widely grown introduced grain legume crops such as lupin and field pea and is found in some introduced weeds [22], [23], [43], [51], [52].

Our findings highlight the need to consider establishment of worldwide conservation policies and management approaches that protect endangered plant species and biodiversity from virus invasion. Such policies and approaches require managing virus disease in situations where new encounters occur at agro-ecological interfaces or natural vegetation is disturbed, and avoiding virus spread to undisturbed native vegetation. Producing them will become increasingly important as new encounters and natural vegetation disturbance are both projected to increase considerably in the future due to climate change induced alterations in the distribution of crops and agricultural extensification to feed the burgeoning human population [3], [38]. Our findings also emphasise the need to tighten quarantine regulations when commercial planting material is moved from one part of the world to another to avoid introducing viruses that may damage natural vegetation [87].

## Supporting Information

Table S1 **Losses in biomass and fruit production in Solanum symonii caused by systemic infection with three viruses.**
(DOCX)
