# Correction: Effects of Introduced and Indigenous Viruses on Native Plants: Exploring Their Disease Causing Potential at the Agro-Ecological Interface

**DOI:** 10.1371/journal.pone.0101542

**Published:** 2014-06-23

**Authors:** The PLOS ONE Staff

There are several errors in this article:

The subtitle of this article is incorrect. The correct subtitle is “Virus Threat to Plant Biodiversity”.

Table 5 is incorrect. The common name of *Hovea elliptica* is missing. The authors have provided a corrected version below.

**Table 5:** Native plants in which infection with introduced or unidentified viruses was detected by ELISA tests on samples

Species	Common name	Site location	No. of plants tested (grouping)	No. of positive samples (% infection)						
				AMV	TuYV*	BYMV	CMV	Potyvirus	Tospovirus	Luteovirus
2001 Samples										
**Caesalpiniaceae**										
*Cassia* sp.	-	Calingiri	1 (1)	-	0	1	-	1	0	0
**Fabaceae**										
*Bossiaea eriocarpa*	Common brown pea	Bindoon	85 (5)	-	0	-	-	0	1 (1)	-
*Bossiaea ornata*	Broad-leaf brown pea	The Lakes	5 (1)	-	1	-	-	0	0	-
*Daviesia nudiflora*	-	Quairading	40 (1)	-	1 (3)	-	-	0	0	0
*Gompholobium* sp.	-	Badgingarra	7 (7)	-	0	-	-	1	0	-
*Hovea elliptica*	Tree hovea	Mt Barker	10 (10)	-	0	-	-	1	0	0
*Kennedia eximia*	-	Bindoon	30 (5)	-	0	3 (13)	-	3 (13)	0	0
*Kennedia prostrata*	Scarlet runner	Brookton	15 (5)	-	0	2	-	2	0	0
*Leptosema aphyllum*	Ribbon pea	Carnamah	21 (7)	-	0	-	-	2 (14)	0	0
**Goodeniaceae**										
*Damperia* sp.	-	Woodanilling	10 (1)	-	1 (10)	-	-	0	0	1 (10)
2009 Samples										
Asparagaceae										
*Chamaescilla corymbosa*	Blue squill	Kings Park	12 (1)	0	0	-	0	12 (100)	0	-
**Droseraceae**										
*Drosera* sp.	Sundew	Wooroloo	6 (6)	-	0	1	0	1	0	-
Fabaceae										
*Hovea elliptica*	Tree hovea	Wellard	1	-	-	-	0	0	1**	-
**Haemodoraceae**										
*Anigozanthos* sp.	Kangaroo paw	Manjumup	60 (10)	0	0	-	0	3 (8)	0	-
*Anigozanthos manglesii*	Mangles kangaroo paw	Wooroloo	1	-	-	-	1	0	0	-
**Hemerocallidaceae**										
*Caesia micrantha*	Grass lily	Kings Park	30 (1)	-	-	-	-	14 (47)	-	-
Juncaginaceae										
*Triglochlin sp.*	Arrowgrass	Helena River	20 (1)	-	-	11(55)	-	-	-	-
*Triglochlin sp.*	Arrowgrass	Guildford	20 (1)	-	-	13 (65)	-	-	-	-
*Triglochlin sp.*	Arrowgrass	Kings Park	18 (1)			18 (100)	-	18 (100)		
*Triglochlin sp.*	Arrowgrass	Not recorded	50 (1)	-	0	-	-	47 (94)		
Orchidaceae										
*Caladenia paludosa*	Common swamp spider-orchid	Kings Park+	2	-	-	2	0	2	0	-
*Cymbidium canaliculatum*	Black orchid	Kings Park+	1	-	-	0	0	1	0	-
*Dendrobium* sp.	-	Kings Park+	1	-	-	0	0	1	0	-
*Diuris longifolia*	Common donkey orchid	Kings Park+	3 (1)	-	-	2	0	3	0	-
*Diuris longifolia*	Common donkey orchid	Kings Park+	46 (1)	-	-	12 (26)	0	25 (54)	0	-
*Diuris micrantha*	Dwarf bee orchid	Kings Park+	1	-	-	1	0	1	0	-
*Microtis sp.*	Onion orchid	Kings Park+	1	-	-	1	0	1	0	-
*Thelymitra sp.*	Sun orchid	Kings Park+	1	-	-	1	0	1	0	-

For an explanation of virus acronyms see Table 1,

- =  Not tested,

* =  TuYV detected by BWYV polyclonal antibodies.

** =  Tospovirus positive sample tested negative for TSWV and *Impatiens necrotic spot virus* (INSV),

+ =  Native orchid collection. Samples were either tested individually or grouped (in 5’s-10’s) before testing. When sufficient grouped samples were present, percentage infection was calculated using the formula of Gibbs and Gower [71]. All orchid samples also tested for *Cymbidium mosaic virus* and *Odontoglossum ringspot virus* by ELISA, but none contained them.

In the Discussion section, the second sentence of the seventh paragraph is incorrect. The correct sentence is, “Previously, seven introduced viruses were reported naturally infecting native plants in the SWAFR (see Introduction).”
