# Supplementary material for: Effects of Introduced and Indigenous Viruses on Native Plants: Exploring Their Disease Causing Potential at the Agro-Ecological Interface
Source: PLoS One. 2014 Mar 12;9(3):e91224. doi: 10.1371/journal.pone.0091224 (PMC3951315; doi:10.1371/journal.pone.0091224)
Supplement: Table S1 — Losses in biomass and fruit production in Solanum symonii caused by systemic infection with three viruses. (DOCX) [file pone.0091224.s001.docx]

**Table S5.** Losses in biomass and fruit production of *Solanum symonii* caused by systemic infection with three generalist introduced viruses^A^

| Expt | Treatment | Mean shoot dry weight (g) | Loss (%) | Mean individual fruit dry weight (g) | Loss  (%) |
| --- | --- | --- | --- | --- | --- |
| 1 | Healthy | 113.9 c | - | 1.5 c | - |
|  | TSWV-infected | 6.4 a | 94 | 0.01 a | 100 |
|  | CMV-infected | 86.3 b,c | 24 | 0.9 b | 40 |
|  | AMV+CMV-infected | 55.4 b | 51 | 0.8 b | 47 |
|  | Residual Df | 7 |  | 7 |  |
|  | Lsd | 32.58 |  | 0.59 |  |
|  | *P* | <0.001 |  | 0.004 |  |
| 2 | Healthy | 35.9 c | - | - | - |
|  | AMV-infected | 23.6 b | 34 | - | - |
|  | TSWV-infected | 10.5 a | 71 | - | - |
|  | Residual df | 8 |  |  |  |
|  | Lsd | 9.24 |  |  |  |
|  | *P* | <0.001 |  |  |  |

Plants infected by sap inoculation of leaves. Treatments arranged in a randomised block design on the glasshouse bench. Tip leaf samples from each plant were tested repeatedly for virus infection by ELISA during each experiment. Dry weight data are means for individual plants or means for individual fruits within a treatment. Values with matching letters next to them were not significantly different from each other. Values for fruit dry weight yields/plant were not significantly different from each other.

^A^ Duration between inoculation and biomass/fruit harvest was 229 (Expt 1) and 198 (Expt 2) days.
